# INTERSECTION OF GENDER, PHYSICAL DISABILITY, AND EXPERIENCES OF ELDER MISTREATMENT IN DEMENTIA

**DOI:** 10.1093/geroni/igae098.0497

**Published:** 2024-12-31

**Authors:** Carolyn Pickering, Wesley Browning, Vicki Winstead, Jessica Hernandez, Mustafa Yildiz

**Affiliations:** The University of Texas Health Houston, Houston, Texas, United States; The University of Texas Health Science Center Houston, Houston, Texas, United States; The University of Texas Health Science Center, Houston, Texas, United States; The University of Texas Health Science Center, Houston, Texas, United States; The University of Texas Health Houston, Houston, Texas, United States

## Abstract

A gerofeminist perspective highlights the intersection of gender, age, and power dynamics that influence the experience of aging women, while recognizing that age can also come with beneficial resources. Since gender-based power dynamics play significant roles in women’s experiences of aggression at younger ages, this study investigates the intersection of gender, physical disability, and elder mistreatment among older adults with dementia. Family caregivers provided self-reports on their care recipients’ characteristics and the frequency of engaging in physically aggressive, psychologically aggressive, and neglectful behaviors at three time points (enrollment, 6-, and 12-month follow-ups). We utilized mixed models for repeated measures to assess whether women experienced more aggressive and neglectful behaviors than men and whether physical disability heightened this risk. Care recipients (N=453) were more often women (58%). Women care recipients required significantly more assistance with ADLs (p<0.001), were more often wheelchair or bedbound (p<0.001) and lived in larger (p=0.003) and more multigenerational households compared to men (p=0.03). Gender-only models revealed that women experienced significantly less neglect than men (p=0.02). When physical disability was considered, wheelchair or bedbound women experienced significantly more neglect (p<0.001) and physical aggression (p<0.001) than men or non-physically disabled women. Findings support a gerofeminist view that women accumulate strengths as they age, such as larger social networks, that may protect against neglect. Findings also suggest that older women with physical disabilities face heightened risks of mistreatment, underscoring the importance of further research into the intersectionality of gender and disability.

